# Supplementary material for: Disinfection exhibits systematic impacts on the drinking water microbiome
Source: Microbiome. 2020 Mar 20;8:42. doi: 10.1186/s40168-020-00813-0 (PMC7085177; doi:10.1186/s40168-020-00813-0)
Supplement: Supplementary file 2 — Additional file 1: Table S1. Summary of water quality parameters measured for the samples collected as part of this study. (*NM = not measured). Table S2. BioEnv analyses in vegan package to determine the subset of variables significantly correlated with community similarities. This determines the Spearman’s correlation between Euclidean distances of scaled environmental variables with the Mash distances estimated using metagenomic reads. Table S3. Distance based Redundancy Analysis using Mash distance matrix generated using pairwise Mash distances between samples estimated using metagenomic reads. Table S4. Variance Partition analysis using water chemistry/environmental parameters identified as significant being significantly associated with read-based Mash distances by dbRDA analyses. Table S5. Number of predicted open reading frames (ORFs) for each metagenome co-assembly and number annotated against the KEGG database using Kofamscan. Table S6. BioEnv analyses in vegan package to determine the subset of variables significantly correlated with similarities in functional potential of community estimates using KEGG annotation. This determines the Spearman’s correlation between Euclidean distances of scaled environmental variables with Bray Curtis distance matrix generated from RPKM of KO detected in samples. Table S7. Distance based Redundancy Analysis using pairwise Bray-Curtis distances between samples estimated using from RPKM of KO detected in samples. Table S8. Variance Partition analysis using water chemistry/environmental parameters identified as significant being significantly associated with KO Bray-Curtis distance matrix by dbRDA analyses. Table S9. Summary of modules that were significantly higher abundance in disinfected systems as compared to non-disinfected systems. Table S10. Summary of modules that were significantly higher abundance in non-disinfected systems as compared to disinfected systems. [file 40168_2020_813_MOESM1_ESM.pdf]

Table S1: Summary of water quality parameters measured for the samples collected as part of this study. (*NM = not measured)

Sample	DWS	Type	Country	Temp	pH	Conductivity	DO	Total Chlorine	Phosphate	TOC	Ammonia	Nitrate
				(ºC)		(mS/cm)	(mg/l)	(mg Cl_2/l)	(mg PO_4^{3-}/l)	(mg/l)	(mg N/l)	(mg N/l)
D1.1	D1	Dis	UK	16.60	8.54	128.90	4.99	0.36	1.09	2.29	0.02	0.60
D1.2	D1	Dis	UK	18.30	8.51	116.50	5.07	0.31	0.98	2.15	0.04	0.48
D1.3	D1	Dis	UK	16.60	8.47	113.00	5.41	0.10	1.12	1.94	0.03	0.42
D1.4	D1	Dis	UK	18.30	8.39	114.50	5.54	0.13	1.15	2.29	0.03	0.49
D2.1	D2	Dis	UK	13.30	8.39	65.50	10.76	0.73	2.44	2.64	0.08	0.21
D2.2	D2	Dis	UK	12.50	8.45	65.00	10.05	0.42	2.06	2.62	0.08	0.17
D2.3	D2	Dis	UK	12.30	8.42	66.00	10.10	0.43	2.10	2.72	0.08	0.37
D2.4	D2	Dis	UK	14.50	8.33	68.70	11.36	0.33	2.07	2.44	0.07	0.25
D3.1	D3	Dis	UK	19.90	7.88	144.75	10.37	0.16	1.23	1.39	0.00	1.49
D3.2	D3	Dis	UK	10.60	7.54	148.25	11.90	0.26	1.82	1.91	0.00	1.53
D4.1	D4	Dis	UK	15.10	7.56	313.00	8.92	0.66	1.97	1.80	0.00	1.10
D4.2	D4	Dis	UK	17.40	7.67	312.50	9.46	0.50	2.00	1.60	0.00	1.01
D4.3	D4	Dis	UK	18.30	7.95	311.50	9.17	0.60	2.12	1.63	0.00	1.07
D4.4	D4	Dis	UK	17.90	7.93	306.00	9.59	0.36	1.96	1.58	0.00	1.00
D5.1	D5	Dis	UK	19.20	7.49	666.00	8.11	0.63	4.94	2.32	0.14	6.07
D5.2	D5	Dis	UK	18.20	7.54	683.50	13.26	0.42	0.85	2.24	0.00	6.02
D5.3	D5	Dis	UK	21.10	7.92	652.00	8.86	0.36	4.67	3.61	0.13	5.68
D5.4	D5	Dis	UK	21.60	7.47	653.00	8.09	0.42	4.77	2.28	0.00	5.82
D6.1	D6	Dis	UK	17.90	7.88	216.40	9.68	0.38	3.06	1.00	0.00	1.70
D6.2	D6	Dis	UK	19.40	8.08	212.60	9.13	0.12	3.05	0.94	0.00	1.64
D6.3	D6	Dis	UK	19.70	8.02	227.65	9.33	0.26	3.09	1.08	0.00	1.73
D8.1	D8	Dis	UK	15.10	8.74	103.35	10.90	0.41	2.48	1.59	0.01	0.27
D8.2	D8	Dis	UK	12.65	8.50	101.15	10.93	0.11	2.37	1.48	0.01	0.20
ND1.1	ND1	NonDis	NL	11.40	8.25	542.00	8.98	0.00	0.03	1.01	0.00	0.62
ND1.2	ND1	NonDis	NL	11.80	8.23	546.00	8.74	0.00	0.03	1.01	0.00	0.60
ND1.3	ND1	NonDis	NL	12.00	8.24	546.00	8.70	0.00	0.03	1.09	0.00	0.61
ND1.4	ND1	NonDis	NL	14.80	NM	NM	NM	0.00	0.03	1.04	0.00	0.55
ND2.1	ND2	NonDis	NL	12.40	8.11	467.00	8.41	0.00	0.00	2.80	0.00	0.99
ND2.2	ND2	NonDis	NL	11.80	8.13	472.00	8.33	0.00	0.03	2.72	0.00	0.91
ND2.3	ND2	NonDis	NL	NM	NM	NM	NM	0.00	0.00	2.63	0.00	0.93
ND3.1	ND3	NonDis	NL	11.70	8.09	619.00	8.50	0.00	0.00	5.60	0.00	2.78

ND3.2	ND3	NonDis	NL	11.30	8.06	599.00	8.50	0.00	0.00	5.50	0.00	2.84
ND3.3	ND3	NonDis	NL	10.70	7.97	618.00	9.10	0.00	0.00	5.40	0.00	2.76
ND3.4	ND3	NonDis	NL	10.10	8.06	614.00	8.70	0.00	0.00	5.40	0.00	2.98
ND4.1	ND4	NonDis	NL	11.90	8.37	322.00	10.20	0.00	0.00	1.00	0.00	0.48
ND4.2	ND4	NonDis	NL	11.40	8.28	336.00	10.30	0.00	0.00	1.10	0.00	0.53
ND4.3	ND4	NonDis	NL	10.80	8.38	334.00	9.70	0.00	0.00	1.00	0.00	0.48
ND5.1	ND5	NonDis	NL	11.60	7.59	507.00	8.60	0.00	0.00	3.90	0.00	2.59
ND5.2	ND5	NonDis	NL	10.30	8.01	527.00	9.30	0.00	0.00	3.80	0.00	0.96
ND5.3	ND5	NonDis	NL	10.70	7.61	482.00	8.70	0.00	0.00	4.20	0.00	2.79
ND5.4	ND5	NonDis	NL	9.50	7.53	506.00	7.50	0.00	0.00	3.20	0.00	2.80

Table S2: BioEnv analyses in vegan package to determine the subset of variables significantly correlated with community similarities. This determines the Spearman's correlation between Euclidean distances of scaled environmental variables with the Mash distances estimated using metagenomic reads.

Number of parameters	Parameter combination	Spearman's correlation	p value
1	Chlorine	0.54	0.001
2	Chlorine + Phosphate	0.58	0.001
3	Chlorine + Phosphate + TOC	0.62	0.001
4	Temp + Chlorine + Phosphate + TOC	0.61	0.001
5	Temp + Conductivity + Chlorine + Phosphate + TOC	0.61	0.001
6	Temp + pH + Conductivity + Chlorine + Phosphate + TOC	0.56	0.001
7	Temp + pH + Conductivity + Chlorine + Phosphate + TOC + Ammonia	0.51	0.001
8	Temp + pH + Conductivity + Chlorine + Phosphate + TOC + Ammonia + Nitrate	0.47	0.001
9	Temp + pH + Conductivity + DO + Chlorine + Phosphate + TOC + Ammonia + Nitrate	0.40	0.001

Table S3: Distance based Redundancy Analysis using Mash distance matrix generated using pairwise Mash distances between samples estimated using metagenomic reads.

Permutation test for dbrda under reduced model
Marginal effects of terms
Permutation: free
Number of permutations: 9999
Model: dbrda(formula = Mash_distance.dist ~ pH + Conductivity + Chlorine + Temp + DO + Phosphate + TOC + Ammonia + Nitrate, data = meta_table[, selected_environmental_data, drop = F])

Variables	Degrees of Freedom	Sum of Squares	F	Pr (>F)	Significance
pH	1	0.028051	2.5851	0.0518	.
Conductivity	1	0.061962	5.7103	0.0029	**
Chlorine	1	0.036368	3.3516	0.021	*
Temperature	1	0.026548	2.4466	0.0593	.
Dissolved oxygen	1	0.033352	3.0737	0.0313	*
Phosphate	1	0.013299	1.2256	0.2787	
Temperature	1	0.020983	1.9338	0.1078	
Ammonia	1	0.013206	1.217	0.2704	
Nitrate	1	0.023434	2.1596	0.0836	.
Residual	28	0.303824			

Signif. codes: 0 '***' 0.001 '**' 0.01 '*' 0.05 '.' 0.1 ' ' 1

Table S4: Variance Partition analyses using water chemistry/environmental parameters identified as significant being significantly associated with read-based Mash distances by dbRDA analyses.

Command used:
Mash_var<-varpart(Mash.dist, ~Chlorine, ~Conductivity, ~DO, data = metadata)

No. of explanatory tables: 3
Total variation (SS): 0.96648
No. of observations: 38

X1 = Chlorine, X2 = Conductivity, X3 = Dissolved oxygen (DO)

**Variables and their combinations**	**Degrees of freedom**	**R square**	**Adjusted R square**	**Testable**
[a+d+f+g] = X1	1	0.29	0.27	TRUE
[b+d+e+g] = X2	1	0.23	0.21	TRUE
[c+e+f+g] = X3	1	0.04	0.02	TRUE
[a+b+d+e+f+g] = X1+X2	2	0.42	0.38	TRUE
[a+c+d+e+f+g] = X1+X3	2	0.31	0.28	TRUE
[b+c+d+e+f+g] = X2+X3	2	0.27	0.23	TRUE
[a+b+c+d+e+f+g] = All	3	0.45	0.40	TRUE
Individual fractions				
[a] = X1|X2+X3	1		0.17	TRUE
[b] = X2|X1+X3	1		0.12	TRUE
[c] = X3|X1+X2	1		0.01	TRUE
[d]	0		0.09	FALSE
[e]	0		-0.01	FALSE
[f]	0		0.01	FALSE
[g]	0		0.00	FALSE
[h] = Residuals			0.60	FALSE
Controlling 1 table X				
[a+d] = X1|X3	1		0.26	TRUE
[a+f] = X1|X2	1		0.17	TRUE
[b+d] = X2|X3	1		0.21	TRUE
[b+e] = X2|X1	1		0.12	TRUE
[c+e] = X3|X1	1		0.01	TRUE
[c+f] = X3|X2	1		0.02	TRUE

Table S5: Number of predicted open reading frames (ORFs) for each metagenome co-assembly and number annotated against the KEGG database using Kofamscan.

DWS	Total predicted ORF's	ORF's annotated using KEGG database	Percent ORF's identified using KEGG database
D1	863400	149048	17.26
D2	75290	20440	27.15
D3	374097	82883	22.16
D4	269598	61517	22.82
D5	410971	85531	20.81
D6	86083	21343	24.79
D8	159482	42639	26.74
ND1	851157	172437	20.26
ND2	571361	102030	17.86
ND3	1034340	205383	19.86
ND4	261064	56331	21.58
ND5	3067778	606300	19.76

Table S6: BioEnv analyses in vegan package to determine the subset of variables significantly correlated with similarities in functional potential of community estimates using KEGG annotation. This determines the Spearman's correlation between Euclidean distances of scaled environmental variables with Bray Curtis distance matrix generated from RPKM of KO detected in samples.

Number of parameters	Parameter combination	Spearman's correlation	p value
1	Chlorine	0.382	0.001
2	Chlorine + Ammonia	0.381	0.001
3	Chlorine + Phosphate + Ammonia	0.392	0.001
4	Conductivity + Chlorine + Phosphate + Ammonia	0.369	0.001
5	Temp + Conductivity + Chlorine + Phosphate + Ammonia	0.361	0.001
6	Temp + Conductivity + Chlorine + Phosphate + Ammonia + Nitrate	0.346	0.001
7	Temp + pH + Conductivity + Chlorine + Phosphate + Ammonia + Nitrate	0.320	0.001

Table S7: Distance based Redundancy Analysis using pairwise Bray-Curtis distances between samples estimated using from RPKM of KO detected in samples.

Permutation test for dbrda under reduced model
Marginal effects of terms
Permutation: free
Number of permutations: 9999
Model: dbrda(formula = BC_distance ~ pH + Conductivity + Chlorine + Temp + DO + Phosphate + TOC + Ammonia + Nitrate, data = meta_table[, selected_environmental_data, drop = F])

Variables	Degrees of Freedom	Sum of Squares	F	Pr (>F)	Significance
pH	1	0.07373	1.1766	0.2907	
Conductivity	1	0.18276	2.9167	0.0184	*
Chlorine	1	0.14964	2.388	0.0412	*
Temperature	1	0.08612	1.3743	0.2053	
Dissolved oxygen	1	0.11357	1.8124	0.1026	
Phosphate	1	0.02585	0.4126	0.8905	
Temperature	1	0.06202	0.9897	0.3816	
Ammonia	1	0.07035	1.1227	0.3101	
Nitrate	1	0.08086	1.2904	0.23	
Residual	28	1.75452			

Signif. codes: 0 '***' 0.001 '**' 0.01 '*' 0.05 '.' 0.1 ' ' 1

Table S8: Variance Partition analyses using water chemistry/environmental parameters identified as significant being significantly associated with KO Bray-Curtis distance matrix by dbRDA analyses.
Command used: KO_var<-varpart(KO.dist, ~Chlorine, ~Conductivity, data=metadata)
No. of explanatory tables: 2
Total variation (SS): 3.2891
No. of observations: 38
X1 = Chlorine, X2 = Conductivity

Variables and their combinations	Degrees of freedom	R square	Adjusted R square	Testable
[a+b] = X1	1	0.11454	0.08994	TRUE
[b+c] = X2	1	0.11503	0.09044	TRUE
[a+b+c] = X1+X2	2	0.20149	0.15586	TRUE
Individual fractions				
[a] = X1|X2	1		0.06542	TRUE
[b]	0		0.02452	TRUE
[c] = X2|X1	1		0.06592	TRUE
[d] = Residuals			0.84414	FALSE

Table S9: Summary of modules that were significantly higher abundance in disinfected systems as compared to non-disinfected systems.

Module	baseMean	log2 FoldChange	lfcSE	stat	pvalue	padj	Module description	Subcategory	Category
M00136	234.06	-27.00	2.95	-9.16	5.03E-20	7.36E-19	GABA biosynthesis, prokaryotes, putrescine => GABA	Polyamine biosynthesis	Nucleotide and amino acid metabolism
M00548	209.34	-26.85	2.95	-9.11	8.00E-20	1.09E-18	Benzene degradation, benzene => catechol	Aromatics degradation	Secondary metabolism
M00367	202.26	-26.54	2.95	-9.01	2.15E-19	2.75E-18	C10-C20 isoprenoid biosynthesis, non-plant eukaryotes	Terpenoid backbone biosynthesis	Carbohydrate and lipid metabolism
M00539	135.17	-26.26	2.95	-8.91	5.11E-19	5.82E-18	Cumate degradation, p-cumate => 2-oxopent-4-enoate + 2-methylpropanoate	Aromatics degradation	Secondary metabolism
M00551	146.57	-25.63	2.37	-10.82	2.90E-27	5.40E-26	Benzoate degradation, benzoate => catechol / methylbenzoate => methylcatechol	Aromatics degradation	Secondary metabolism
M00861	4.30	-21.58	2.95	-7.32	2.57E-13	2.03E-12	beta-Oxidation, peroxisome, VLCFA	Fatty acid metabolism	Carbohydrate and lipid metabolism
M00107	3339.85	-5.93	1.35	-4.39	1.14E-05	6.32E-05	Steroid hormone biosynthesis, cholesterol => prognenolone => progesterone	Sterol biosynthesis	Carbohydrate and lipid metabolism
M00545	7618.51	-4.83	1.27	-3.80	1.43E-04	6.66E-04	Trans-cinnamate degradation, trans-cinnamate => acetyl-CoA	Aromatic amino acid metabolism	Nucleotide and amino acid metabolism
M00597	7007.83	-3.87	1.04	-3.71	2.04E-04	9.07E-04	Anoxygenic photosystem II	Photosynthesis	Energy metabolism
M00568	4616.59	-3.83	0.91	-4.23	2.35E-05	1.27E-04	Catechol ortho-cleavage, catechol => 3-oxoadipate	Aromatics degradation	Secondary metabolism
M00027	7825.16	-3.73	1.07	-3.50	4.73E-04	1.98E-03	GABA (gamma-Aminobutyrate) shunt	Other amino acid metabolism	Nucleotide and amino acid metabolism
M00061	13554.83	-3.25	0.82	-3.96	7.57E-05	3.78E-04	D-Glucuronate degradation	Other carbohydrate metabolism	Carbohydrate and lipid metabolism

M00631	12841.60	-2.40	0.75	-3.21	1.34E-03	4.82E-03	D-Galacturonate degradation (bacteria)	Other carbohydrate metabolism	Carbohydrate and lipid metabolism
M00066	15629.00	-2.09	0.54	-3.88	1.03E-04	5.03E-04	Lactosylceramide biosynthesis	Lipid metabolism	Carbohydrate and lipid metabolism
M00741	25451.60	-1.98	0.60	-3.31	9.29E-04	3.73E-03	Propanoyl-CoA metabolism, propanoyl-CoA => succinyl-CoA	Other carbohydrate metabolism	Carbohydrate and lipid metabolism
M00307	27139.54	-1.69	0.42	-4.06	4.88E-05	2.56E-04	Pyruvate oxidation, pyruvate => acetyl-CoA	Central carbohydrate metabolism	Carbohydrate and lipid metabolism
M00036	24198.24	-1.62	0.40	-4.03	5.59E-05	2.86E-04	Leucine degradation, leucine => acetoacetate + acetyl-CoA	Branched-chain amino acid metabolism	Nucleotide and amino acid metabolism
M00088	35331.02	-1.60	0.30	-5.32	1.04E-07	6.87E-07	Ketone body biosynthesis, acetyl-CoA => acetoacetate/3-hydroxybutyrate/acetone	Fatty acid metabolism	Carbohydrate and lipid metabolism
M00011	33315.58	-1.12	0.14	-8.00	1.26E-15	1.17E-14	Citrate cycle, second carbon oxidation, 2-oxoglutarate => oxaloacetate	Central carbohydrate metabolism	Carbohydrate and lipid metabolism
M00009	33466.36	-0.86	0.15	-5.57	2.52E-08	1.78E-07	Citrate cycle (TCA cycle, Krebs cycle)	Central carbohydrate metabolism	Carbohydrate and lipid metabolism
M00173	25002.47	-0.83	0.23	-3.66	2.52E-04	1.10E-03	Reductive citrate cycle (Arnon-Buchanan cycle)	Carbon fixation	Energy metabolism
M00121	46144.04	-0.57	0.11	-5.13	2.93E-07	1.88E-06	Heme biosynthesis, glutamate => heme	Cofactor and vitamin biosynthesis	Nucleotide and amino acid metabolism

Table S10: Summary of modules that were significantly higher abundance in non-disinfected systems as compared to disinfected systems.

Module	baseMean	log2 FoldChange	lfcSE	stat	pvalue	padj	Module description	Subcategory	Category
M00761	1255.12	30.00	1.46	20.55	7.30E-94	7.48E-92	Undecaprenylphosphate alpha-L-Ara4N biosynthesis, UDP-GlcA => undecaprenyl phosphate alpha-L-Ara4N	Other carbohydrate metabolism	Carbohydrate and lipid metabolism
M00159	885.38	30.00	1.45	20.71	2.92E-95	5.98E-93	V-type ATPase, prokaryotes	ATP synthesis	Energy metabolism
M00377	475.90	30.00	2.19	13.67	1.51E-42	4.44E-41	Reductive acetyl-CoA pathway (Wood-Ljungdahl pathway)	Carbon fixation	Energy metabolism
M00620	786.19	30.00	2.11	14.22	7.20E-46	2.46E-44	Incomplete reductive citrate cycle, acetyl-CoA => oxoglutarate	Carbon fixation	Energy metabolism
M00175	4374.13	30.00	2.07	14.47	1.79E-47	7.34E-46	Nitrogen fixation, nitrogen => ammonia	Nitrogen metabolism	Energy metabolism
M00847	164.40	29.94	2.21	13.55	7.99E-42	2.05E-40	Heme biosynthesis, archaea, siroheme => heme	Cofactor and vitamin biosynthesis	Nucleotide and amino acid metabolism
M00422	152.52	29.84	2.24	13.33	1.47E-40	3.36E-39	Acetyl-CoA pathway, CO2 => acetyl-CoA	Methane metabolism	Energy metabolism
M00042	57.83	28.48	2.94	9.69	3.28E-22	5.60E-21	Catecholamine biosynthesis, tyrosine => dopamine => noradrenaline => adrenaline	Aromatic amino acid metabolism	Nucleotide and amino acid metabolism
M00094	59.01	27.78	2.94	9.45	3.34E-21	5.27E-20	Ceramide biosynthesis	Lipid metabolism	Carbohydrate and lipid metabolism
M00836	23.76	27.05	2.39	11.34	8.62E-30	1.77E-28	Coenzyme F430 biosynthesis, sirohydrochlorin => coenzyme F430	Cofactor and vitamin biosynthesis	Nucleotide and amino acid metabolism
M00099	12.71	26.36	2.94	8.96	3.24E-19	3.90E-18	Sphingosine biosynthesis	Lipid metabolism	Carbohydrate and lipid metabolism
M00801	9.91	25.99	2.94	8.83	1.03E-18	1.07E-17	dTDP-L-olivose biosynthesis	Other carbohydrate metabolism	Carbohydrate and lipid metabolism

M00803	9.89	25.99	2.94	8.83	1.05E-18	1.07E-17	dTDP-D-angolosamine biosynthesis	Other carbohydrate metabolism	Carbohydrate and lipid metabolism
M00047	6.09	25.32	2.95	8.59	8.46E-18	8.26E-17	Creatine pathway	Other amino acid metabolism	Nucleotide and amino acid metabolism
M00179	29657.67	19.39	0.98	19.69	2.82E-86	1.92E-84	Ribosome, archaea	Ribosome	Genetic information processing
M00840	20672.63	18.87	0.98	19.28	8.27E-83	4.24E-81	Tetrahydrofolate biosynthesis, mediated by ribA and trpF, GTP => THF	Cofactor and vitamin biosynthesis	Nucleotide and amino acid metabolism
M00031	584.40	11.18	2.05	5.47	4.61E-08	3.15E-07	Lysine biosynthesis, mediated by LysW, 2-aminoadipate => lysine	Lysine metabolism	Nucleotide and amino acid metabolism
M00100	149.70	6.09	1.86	3.27	1.07E-03	4.22E-03	Sphingosine degradation	Lipid metabolism	Carbohydrate and lipid metabolism
M00623	5079.42	5.50	1.51	3.65	2.67E-04	1.14E-03	Phthalate degradation, phthalate => protocatechuate	Aromatics degradation	Secondary metabolism
M00183	107701.24	2.15	0.28	7.59	3.11E-14	2.65E-13	RNA polymerase, bacteria	RNA polymerase	Genetic information processing
M00308	95129.26	2.01	0.33	6.09	1.10E-09	8.39E-09	Semi-phosphorylative Entner-Doudoroff pathway, gluconate => glycerate-3P	Central carbohydrate metabolism	Carbohydrate and lipid metabolism
M00178	105678.14	1.92	0.26	7.33	2.27E-13	1.86E-12	Ribosome, bacteria	Ribosome	Genetic information processing
M00157	98147.17	1.75	0.29	6.00	1.94E-09	1.42E-08	F-type ATPase, prokaryotes and chloroplasts	ATP synthesis	Energy metabolism
M00855	20526.10	1.60	0.50	3.21	1.33E-03	4.82E-03	Glycogen degradation, glycogen => glucose-6P	Other carbohydrate metabolism	Carbohydrate and lipid metabolism
M00141	91291.55	1.56	0.20	7.97	1.55E-15	1.38E-14	C1-unit interconversion, eukaryotes	Cofactor and vitamin biosynthesis	Nucleotide and amino acid metabolism

M00053	75805.14	1.02	0.21	4.81	1.47E-06	8.62E-06	Pyrimidine deoxyribonuleotide biosynthesis, CDP/CTP => dCDP/dCTP,dTDP/dTTP	Pyrimidine metabolism	Nucleotide and amino acid metabolism
M00007	42251.43	0.95	0.19	4.99	6.10E-07	3.79E-06	Pentose phosphate pathway, non-oxidative phase, fructose 6P => ribose 5P	Central carbohydrate metabolism	Carbohydrate and lipid metabolism
M00048	52586.77	0.87	0.19	4.49	7.23E-06	4.12E-05	Inosine monophosphate biosynthesis, PRPP + glutamine => IMP	Purine metabolism	Nucleotide and amino acid metabolism
M00052	85948.16	0.83	0.22	3.84	1.21E-04	5.78E-04	Pyrimidine ribonucleotide biosynthesis, UMP => UDP/UTP,CDP/CTP	Pyrimidine metabolism	Nucleotide and amino acid metabolism
M00168	44967.05	0.73	0.23	3.25	1.17E-03	4.53E-03	CAM (Crassulacean acid metabolism), dark	Carbon fixation	Energy metabolism
M00035	52458.61	0.71	0.21	3.32	8.87E-04	3.64E-03	Methionine degradation	Cysteine and methionine metabolism	Nucleotide and amino acid metabolism
M00002	50244.03	0.68	0.14	4.91	9.09E-07	5.48E-06	Glycolysis, core module involving three-carbon compounds	Central carbohydrate metabolism	Carbohydrate and lipid metabolism
M00125	46145.15	0.54	0.14	3.75	1.76E-04	8.04E-04	Riboflavin biosynthesis, GTP => riboflavin/FMN/FAD	Cofactor and vitamin biosynthesis	Nucleotide and amino acid metabolism
M00049	63394.75	0.49	0.15	3.23	1.22E-03	4.62E-03	Adenine ribonucleotide biosynthesis, IMP => ADP,ATP	Purine metabolism	Nucleotide and amino acid metabolism
M00050	61091.85	0.45	0.14	3.22	1.30E-03	4.82E-03	Guanine ribonucleotide biosynthesis IMP => GDP,GTP	Purine metabolism	Nucleotide and amino acid metabolism

Table S11: Summary statistics for metagenome assembled genomes (MAGs) extracted from the metagenome assemblies. Metagenome assembled genomes (MAG) were finalized after dereplication using dRep (https://github.com/MrOlm/drep) using all MAGs assembled in this study. As a result, the name assigned to a MAG does not represent sampling location it was assembled from. MAGs were assigned taxonomy using the Genome taxonomy database (GTDB-TK: https://gtdb.ecogenomic.org/) version 0.1.3. The completeness and redundancy of MAGs was estimated using CheckM (https://github.com/Ecogenomics/CheckM/wiki) version 1.0.7. Only MAGs >50% completeness and <10% redundancy were included in the study. The genome statistics were estimated using Prokka. The coding density was estimated by dividing the cumulative length of coding sequences (CDS) divided by the length of the MAG. The MAGs were assigned four categories, "D-only", "ND-only", "Both", and "Other". "D-only" was assigned to MAGs detected in >20% of disinfected samples and not detected in non-disinfected samples. "ND-only" was assigned to MAGs detected in <20% of disinfected samples and not detected of non-disinfected samples. "Both" was assigned to MAGs that were detected in >20% of disinfected and non-disinfected samples. "Other" was assigned to MAGs that did not fall in either of the above three classes. (see excel spreadsheet).

Table S12: KEGG modules and their completeness estimates within each MAG assembled as part of this study.
